# Site- and Time-Dependent Compositional Shifts in Oral Microbiota Communities

**DOI:** 10.3389/froh.2022.826996

**Published:** 2022-03-01

**Authors:** Anders Esberg, Linda Eriksson, Ingegerd Johansson

**Affiliations:** Department of Odontology, Umeå University, Umeå, Sweden

**Keywords:** microbiota, oral, tooth biofilm, saliva, sequencing

## Abstract

**Objectives:**

The oral microbiota plays a significant role in oral health. The present study aims to characterize variations in the oral microbiota relative to the collection site, the dynamics of biofilm accumulation, and inherent inter-individual differences.

**Methods:**

Whole stimulated saliva and tooth biofilm samples from the 16 defined tooth regions were collected after 1, 2, or 3 days without oral hygiene (accumulation time) in six healthy adults with no signs of active caries or periodontal disease. The routines and conditions before and between sample collections were carefully standardized. Genomic DNA was extracted, and the V3-V4 regions of the 16S rRNA gene were amplified by PCR and sequenced on an Illumina MiSeq platform. Sequences were quality controlled, amplicon sequence variants (ASVs) were clustered, and taxonomic allocation was performed against the expanded Human Oral Microbiome Database (*e*HOMD). Microbial community profiles were analyzed by multivariate modeling and a linear discriminant analysis (LDA) effect size (LEfSe) method.

**Results:**

The overall species profile in saliva and tooth biofilm differed between participants, as well as sample type, with a significantly higher diversity in tooth biofilm samples than saliva. On average, 45% of the detected species were shared between the two sample types. The microbiota profile changed from the most anterior to the most posterior tooth regions regardless of whether sampling was done after 1, 2, or 3 days without oral hygiene. Increasing accumulation time led to higher numbers of detected species in both the saliva and region-specific tooth biofilm niches.

**Conclusion:**

The present study confirms that the differences between individuals dominate over sample type and the time abstaining from oral hygiene for oral microbiota shaping. Therefore, a standardized accumulation time may be less important for some research questions aiming at separating individuals. Furthermore, the amount of DNA is sufficient if at least two teeth are sampled for microbiota characterization, which allows a site-specific characterization of, for example, caries or periodontitis.

## Introduction

The human body is covered by microorganisms, with the number of bacterial cells slightly outnumbering the number of somatic cells [1]. Among various microbial niches in man, the oral cavity is among the most heavily colonized site, with up to 1,000 microbial species identified [2] and up to 300 bacterial species in a single person at a given time point [3]. Not only the warm and moist environment, epithelial surfaces and hard tooth surfaces, and restorative materials but also ingested foods and drinks, and crevicular fluids and saliva provide a variety of local physicochemical disparities in the oral cavity. These distinctions in preconditions for bacteria to attach and thrive manifests as site-specific eubiotic or dysbiotic multispecies biofilms [4].

Thus, distinct taxonomic and genomic profiles of niche-specific communities in the different parts of the body, but also within the mouth, have been described in both health and disease [5–7]. In the oral cavity, epithelial cell and tooth adhering saliva proteins, glycoproteins, and glycolipids are key players for an initial species-specific attachment of bacteria [8]. Further, innate immunity components and other immune regulating components affect their metabolism and survival [8]. In brief, the initial attachment of bacteria, which are commonly species in the *Streptococcus* and *Actinomyces* genera, are followed by secondary and tertiary colonizers in an interspecies defined manner described as corncob-, hedgehog-, or cauliflower-like structures [9]. Thus, beyond the physicochemical factors mentioned above, the surface-associated biofilms in the oral cavity manifest from bacterial adhesion to the tissue or tissue-associated receptors, interspecies receptor and adhesion interactions, and bacteria cell–cell signaling [8, 10]. One factor that disturbs this sequential building of the biofilms in the oral cavity is tooth brushing and other oral hygiene measures.

Studies on the role of the oral microbiota in dental diseases commonly rely on sequencing DNA from whole saliva or pooled tooth scrapings rather than from the disease location *per se* [7, 11]. Saliva, which is easy to sample, is thought to mirror all tooth and mucosal biofilms [7, 12], but the lack of a more precise niche specificity may be a disadvantage. For example, tooth biofilm pooled from all available tooth surfaces usually means that the majority of the surfaces are healthy and non-representative of caries or periodontitis-affected sites [11]. Tooth-specific sampling, however, entails some challenges; it requires trained personal and is time-consuming, the DNA yield is low and the number of samples becomes large. This may explain why such studies are few and the number of participants is limited.

Several studies have explored oral microbiota temporal stability [13, 14], intra- and inter-individual variations [11], and maturation in the 1st year of life [15] using saliva or tooth scrapings. In the past, most such studies relied on species-specific methods, such as culturing and DNA probes in chip or blotting-based methods, but in recent decades several multiplex sequencing methods that allow the characterization of the concerted microbial communities, i.e., microbiomes have emerged. From such studies, it is proposed that the oral microbiota is highly resilient [14, 16]. However, no study has evaluated the effect of variation in biofilm accumulation time, i.e., lack of oral hygiene, using a multiplex technique. Further, apart from differences between the tongue, other soft tissues, and teeth, regional variations in the mouth are sparsely described. Some studies compared tooth-level microbiota in caries-free and caries diseased children with comparisons stratified for caries status but where the site-specific data may be pooled in the final analyses and some target the pits and fissures only [17–19]. One study by Simon-Soro et al. [20] report tooth-site specificity in two healthy subjects. Their results indicate differences between the different regions of the tooth arches but a few participants restrain the estimation of population variation. And also, a low number of reads per sample may indicate that the DNA yield was low when sampling was restricted to a single tooth surface.

In this study, we aimed to characterize variations in the oral microbiota relative to sampling sites, the dynamics of biofilm accumulation, and inherent inter-individual variations. More precisely, we employed Illumina sequencing of 16S rRNA gene amplicons to assess and compare microbial community profiles in saliva or dental biofilms. Furthermore, biofilm samples collected from the 16 different dental sites were selected to allow the discrimination of anterior vs. posterior and maxillary vs. mandibular regions. Finally, compositional variations in community profiles during biofilm maturation up to 72 h (3 days) and inherent differences between individuals were evaluated.

## Methods

### Study Participants

Eligible participants were informed of this study. Six volunteers (2 men and 4 women), who fulfilled the inclusion criteria of being healthy, having no medication known to reduce the saliva flow rate, no antibiotic treatment for the last 6 months and no probiotics in the last week, a body mass index above 20 and below 25, being a nontobacco user, and having a healthy oral status, consented to participate. Saliva and tooth biofilm were sampled at three occasions. On each occasion, the participants completed a questionnaire on antibiotic and probiotic use, medication, health status, tobacco, and alcohol use 24 h before sampling.

### Oral Examination

A few days before the first sampling, oral status was evaluated by visual and radiographic examinations, including the assessment of tooth restorations, gingival bleeding, signs of caries, pocket depth, attachment loss, and oral mucosa condition.

### Saliva and Supragingival Tooth Biofilm Collection

Saliva and supragingival tooth biofilm samples were collected from all accessible buccal, palatal, lingual, and approximal surfaces after biofilm accumulation, i.e., no tooth brushing or flossing, use of fluoride, chlorhexidine, or any other mouth wash, for 1, 2, and 3 days (accumulation time), with 1 week between the sampling occasions. During the study period, the participants were allowed to maintain their habitual eating but, during the accumulation time, they were instructed to abstain from alcohol, chewing gum, and products containing xylitol or probiotic bacteria. Sample collection took place after 1 p.m., and the participants abstained from eating and drinking for 1 h prior to sample collection. Whole saliva was collected in sterile ice-chilled tubes under paraffin chewing, and tooth biofilm samples were collected at available tooth surfaces using sterile curettes (LM universal Langer 3-4, 283-284 ES and LM universal Gracey 3-4, 203-204 ES, LM Dental, Pargas, Finland). Samples were pooled separately for the molars, premolars, canine, and incisors from the upper and lower jaw and the right and left sides, resulting in a total of 16 regional sample sites per participant and sampling occasion. The curettes were swirled for 10 s in 200 μl of sterile ice-chilled 1 × TE buffer in Eppendorf tubes. All samples were kept on ice until placed in an −80°C freezer within 4 h and stored until DNA extraction. All participants completed all samplings.

### DNA Extraction and Microbiota Analysis

DNA was extracted using the GenElute Bacterial genomic DNA kit (Sigma-Aldrich Co, Stockholm, Sweden) from 400 μl saliva or 200 μl biofilm samples. Briefly, samples were centrifuged for 5 min at 13,000 rpm, lysed in buffer containing lysozyme and mutanolysin, and treated with RNase and Proteinase K, and DNA was purified, washed, and eluted in 150 μl room temperature elution buffer. The quality of the extracted DNA was estimated using a NanoDrop 1000 Spectrophotometer (Thermo Fisher Scientific, Uppsala, Sweden) and the quantity by the Qubit 4 Fluorometer (Invitrogen, Thermo Fisher Scientific, Waltham, MA, USA) of which all samples displayed a concentration >0.5 ng/μl (Supplementary Table S1). The same DNA extraction was applied to Milli-Q Ultrapure water and a mixture of 14 oral bacterial species (mock) serving as negative and positive controls, respectively. Bacteria 16S rDNA amplicons were generated from saliva and tooth biofilm samples, as well as a mock, extracted DNA by fusion PCR of the V3-V4 regions using the primers 341F (ACGGGAGGCAGCAG) and 806R (GGACTACHVGGGTWTCTAAT) as described by Caporaso [21]. Equal amounts of amplicon libraries were pooled and purified using AMPure XP beads (Beckman Coulter, Stockholm, Sweden), followed by Illumina Miseq sequencing, including 5% PhiX (Illumina, Stockholm, Sweden). Three mock samples and two negative controls (ultrapure water) were included in each run. The generated raw sequences were demultiplexed, pair-end reads were fused, and primers, ambiguous, chimeric, and PhiX sequences were removed. Amplicon sequence variants (ASVs) were obtained using DADA2 in the QIIME2 platform (https://qiime2.org) [22, 23]. ASVs were taxonomically classified against the expanded Human Oral Microbiome Database (*e*HOMD) (http://www.homd.org) [23, 24]. ASVs with ≥2 reads and 98.5% identity with a named species or unnamed phylotype in *e*HOMD were retained and aggregated. For readability, both named species and HMT phylotypes are referred to as species in the text. For the 18 mock samples, the median (min, max) number of matched pair-end reads were 22,535 (6,075, 41,594) and detected all 14 including species. For the 12 negative control (Milli-Q ultrapure water), the number of reads were 75 (40, 213) and had <70 reads of highly common taxa and likely reflects some cross-contamination.

### Statistical Analysis

Descriptive statistics are summarized as medians with quartile limits, means with SD, or proportions (%). Aggregated quality-filtered reads were normalized as the proportion of all reads for the sample, and detection (carriage) of a species was set to having ≥2 reads for the species.

Microbiota diversity was assessed as the α-diversity using the number (abundance) or detection of ASVs and the Shannon diversity index (which evaluates abundance and evenness), and as the β-diversity using unweighted Unifrac (based on phylogenetic similarities) and Bray–Curtis dissimilarity (based on species abundances) using QIIME2. All tests were two-sided and values of *p* < 0.05 were considered significant after controlling for multiple testing by the false discovery rate (FDR). For β-diversity measurements, adjusted values of *p* are presented as the FDR-derived *q*-value.

Multivariate orthogonal partial least squares discriminant analysis (OPLS-DA) [25] was used to identify species associated with participants, sample type, tooth section, and accumulation time. All models were fitted using SIMCA P+ version 15.0 (Sartorius Stedim Data Analytics AB, Malmö, Sweden), and all variables were scaled to unit variance and log-transformed. Cross-validation was performed using a *K*-fold method with systematic removal of every 7th observation and prediction by the remaining observations (*Q*^2^ values). The overall model fit [i.e., the sample separation with variance explained (*R*^2^) and variance predicted (*Q*^2^) in the fitted and cross-validated models] is reported in loading scatter plots. Multivariate PLS modeling allows the estimation of both the explanatory and predictive power of many, and even covarying, *X*-variables when modeled simultaneously against outcomes [*Y*-variable(s)]. Variable importance in the projection (VIP) values for generated components reflect the importance of each *X*-variable in explaining the variation in *Y*, i.e., their “correlation” with the *Y*-variable. VIP values are presented for the predictive components only. VIP-values > 1 are considered to be significant and values >1.5 are highly significant. Here, VIP > 1.5 was selected to restrict the selection to species that were highly associated with the specific outcome. Volcano plots based on the VIP values (a metric summary of the significance of each variable in the outcome projection) and *p*(corr) [a loading scale based on the correlation coefficient between the model score (*t*) and the *X*-variable providing a measurement of reliability] were used to identify the most influential species in the model. Ordinal linear regression was used to evaluate ASVs and the Shannon diversity index by anterior and posterior positions (incisor, canine, premolar, and molar tooth positions). A mixed linear model was used to evaluate the number of species, ASVs, and Shannon diversity trends by accumulation time. Anterior vs. posterior and upper vs. lower jaw microbiotas were compared using a non-parametric test and alpha- and beta-group-significance pipelines in QIIME2. The linear discriminant analysis (LDA) effect size (LEfSe) method was used for high-dimensional class comparisons of posterior and anterior parts of the oral cavity, and temporal trends in the tooth biofilm and saliva microbiota [26].

## Results

### Participant and Sequencing Characteristics

The mean (SD) age of the six participants was 26.7 (6.7) years. None reported the use of tobacco, alcohol, or probiotics during the study period. The participants had a healthy oral status, with four being fully dentated and two having had extractions (two teeth each) for orthodontic reasons. None had a probing pocket depth >4 mm, none had any sign of jaw bone loss, and gingival bleeding was present at single sites only. Furthermore, none of the participants had any untreated caries cavities (i.e., manifest caries into the dentin-enamel junction). A few teeth had signs of early non-active enamel caries and a few had a restoration.

A total of 306 samples were evaluated for the microbiota composition by V3-V4 16S rRNA gene sequencing. After demultiplexing, filtering, and paired-end fusion, there were a total of 8,056,613 reads with a median of 23,214 (*Q*_25%_ = 18,600 and *Q*_75%_ = 29,922; mean 23,978) reads per sample. The quality-controlled reads corresponded to 2,070 ASVs, 0.5% of which had no match in *e*HOMD, 19.8% did not meet the criterion of 98.5% identity with a sequence in the *e*HOMD 16S rRNA gene database, and 79.7% (*n* = 1,649 ASVs) matched a sequence at 98.5% (or higher) identity. The 1,649 ASVs represented 297 species in 79 genera and 11 phyla.

### Individual Fingerprint From Tooth Biofilm and Saliva Microbiota Pattern

**Figure 1:**
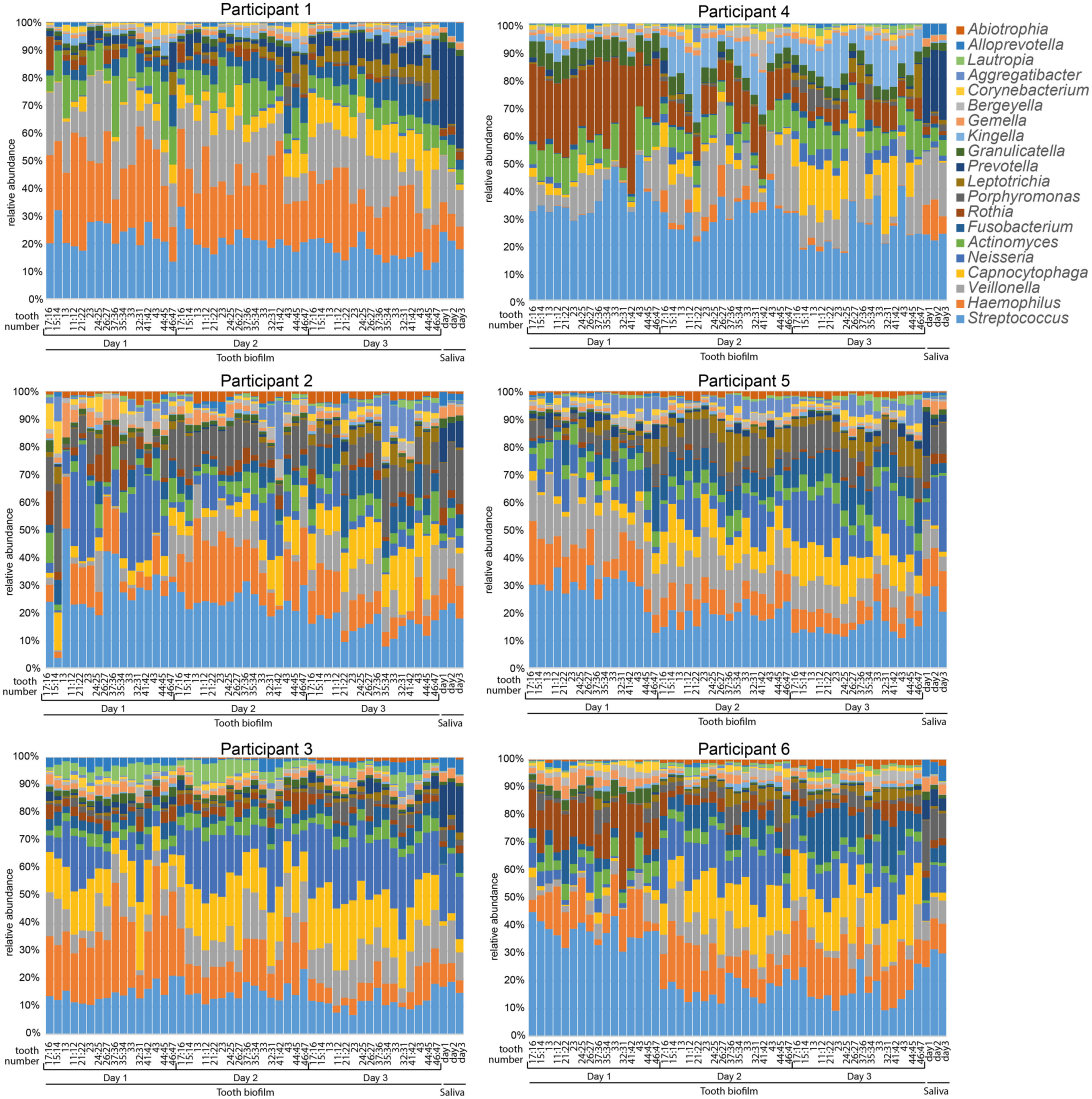
Stacked bar graphs illustrating microbiota profiles in saliva and 16 selected tooth regions after 1, 2, or 3 days without oral hygiene (accumulation time). Data are shown for each of the six participants and the labels at the bottom of the figures indicate sample type, teeth sampled for the 16 tooth regions, and days without oral hygiene. Tooth numbers are given according to the FDI World Dental Federation notation system. The *y*-axis shows the relative proportion (%) for the top 20 detected genera. The colors in the figures match the colors for the genus in the list to the right.

The overall relative microbiota composition at the 16 tooth sites and in saliva after 1, 2, or 3 days without oral hygiene (accumulation time) for each of the six participants is shown in Figure 1. To further evaluate the individual site-specific microbiota fingerprint in the oral cavity, the tooth biofilm and saliva samples were characterized and compared in OPLS-DA models with sample type, sample region, and accumulation time as dependent variables and species abundance as the independent variables as described in each section.

**Figure 2:**
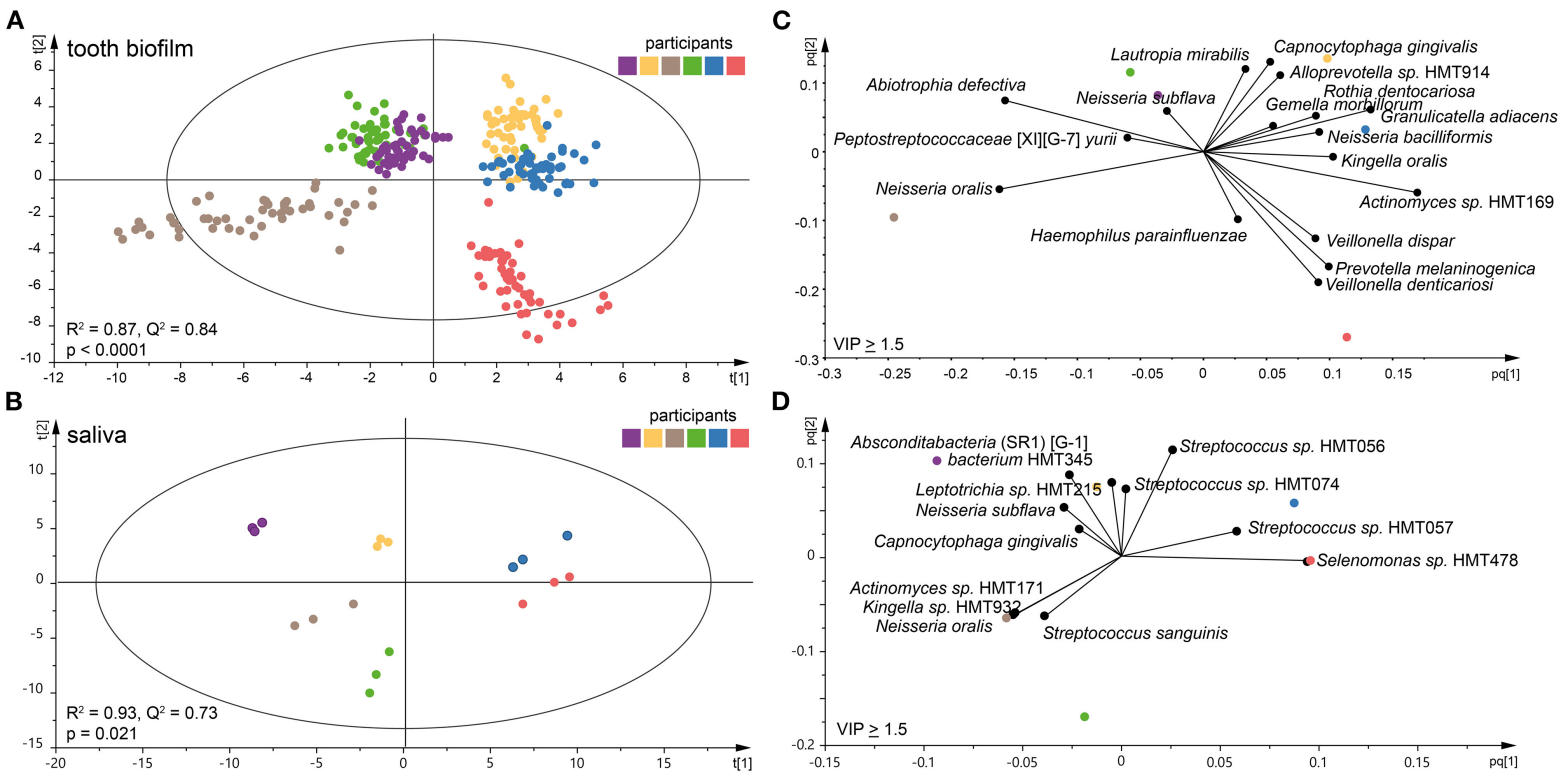
Clustering of the six participants by tooth biofilm and saliva microbiota samples. Orthogonal partial least squares discriminant analysis (OPLS-DA) derived score scatter plots for **(A)** tooth biofilm samples from all 16 tooth regions and three accumulation times and **(B)** saliva samples from three accumulation times. The plots show group separation by discrimination component 1 (*t* [1]) and 2 (*t* [2]). OPLS-DA derived loading scatter bi-plots for **(C)** tooth biofilm and **(D)** saliva in which the *x*-loading [*p*] and *y*-loading [*q*] for the first [*pq*1] and second [*pq*2] predictive components are superimposed.

Species abundance profiles in pooled tooth biofilm samples (i.e., from the 16 tooth sites at three accumulation times) vs. pooled saliva (i.e., from the three accumulation times) formed distinct clusters for each of the six participants (Figures 2A,B), supporting the individual patterns seen at the genus level in Figure 1. For tooth biofilm samples, a strong model (*R*^2^ = 0.87, *Q*^2^ = 0.84) materialized a participant-unique microbiota profile that overshadowed accumulation time and tooth region (Figure 2A). The associated score scatter bi-plot specified *Abiotrophia defective, Peptostreptococcaceae* [XI][G-7] *yurii, Neisseria oralis, Neisseria subflava, Lautropia mirabilis, Haemophilus parainfluenzae, Capnocytophaga gingivalis, Alloprevotella* sp. HMT914, *Rothia dentocariosa, Gemella morbillorum, Granulicatella adiacens, Neisseria bacilliformis, Kingella oralis, Actinomyces* sp. HMT169, *Veillonella dispar, Prevotella melaninogenica*, and *Veillonella denticariosi* as strongly influential (VIP > 1.5) in participant separation (Figure 2C).

For saliva, the OPLS-DA model revealed an equally strong model (*R*^2^ = 0.93, *Q*^2^ = 0.73) with distinct participant clustering (Figure 2B). Here, the score scatter bi-plot of highly influential species (VIP > 1.5, *n* = 12) specified *Absconditabacteria* (SR1) [G-1] *bacterium* HMT345, *Leptotrichia* sp. HMT215, *N. subflava, C. gingivalis, Actinomyces* sp. HMT171, *Kingella* sp. HMT932, *N. oralis, Streptococcus sanguinis, Streptococcus* sp. HMT056, *Streptococcus* sp. HMT057, *Streptococcus* sp. HMT074, and *Selenomonas* sp. HMT478 as important for participant separation (Figure 2D).

### Characteristics of Tooth Biofilm vs. Saliva Microbiota

**Figure 3:**
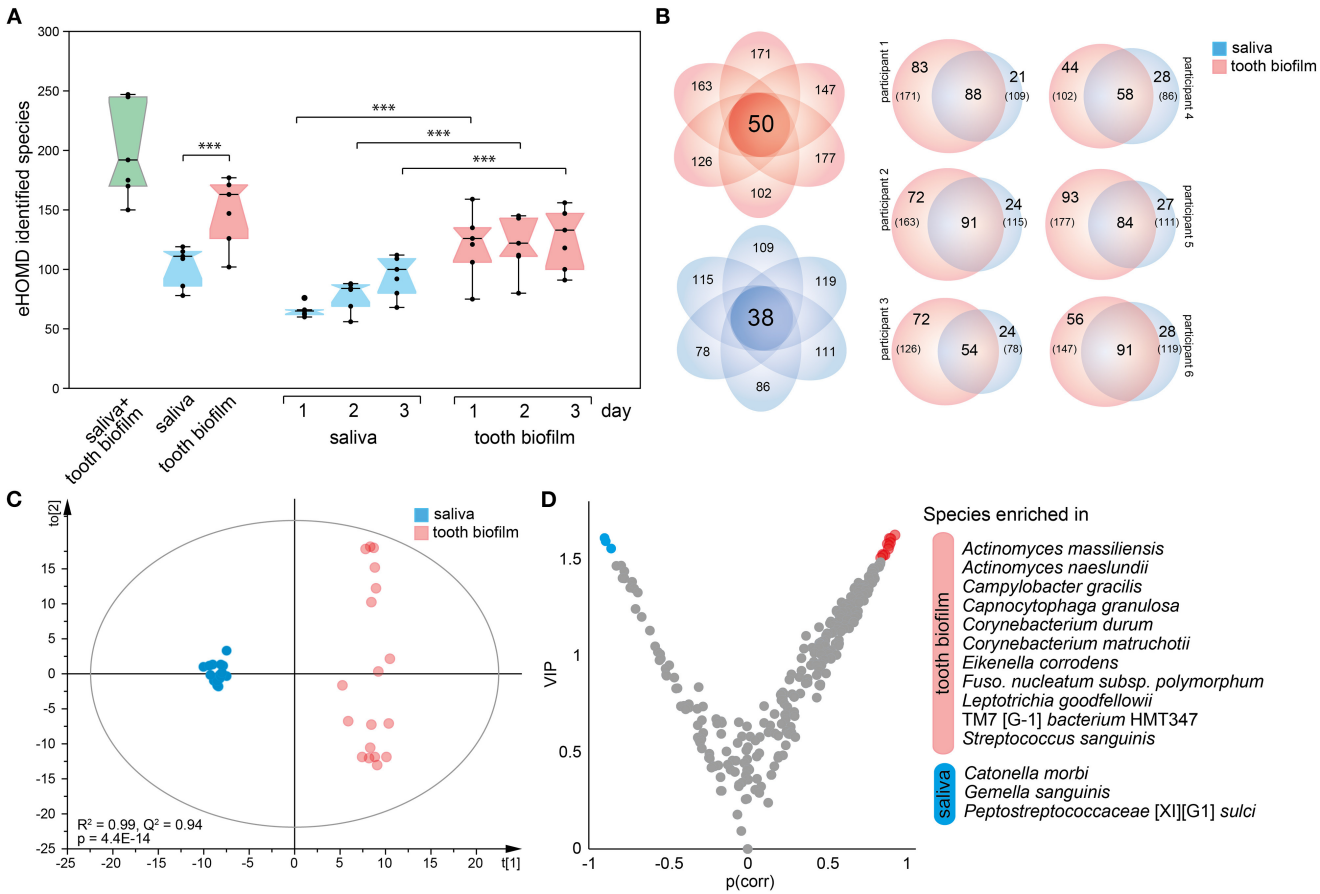
Characteristics of tooth biofilm vs. the saliva microbiota. **(A)** Box plots showing the median with 25 and 75% percentile limits for the number of species in the pan-microbiota, and for the tooth biofilm pooled for all 16 tooth regions, and saliva, respectively. ^***^p < 0.001. **(B)** Venn diagrams showing saliva (blue) and tooth biofilm (red) overlapping species at any accumulation time in (left) all participants or (right) each of the six participants. **(C)** OPLS-DA derived score scatter plot illustrating the clustering of samples from saliva vs. tooth biofilm based on species abundance profiles in the two sample types. The plot of discrimination component *t*[1] vs. orthogonal component to [1] confirms the separation of saliva and tooth biofilm microbiotas in each of the participants and shows that tooth biofilm samples exhibit greater within-subgroup diversity that is not linked to the discrimination component. **(D)** Volcano plot of *p*(corr) (PLS correlation coefficient > 0.5) vs. variable influence in projection (VIP) values (>1.5) highlighting the most influential bacterial species in the separation of the two sample types.

The per-participant number of detected species in the pan-oral microbiota, i.e., pooled tooth biofilm and saliva data, ranged from 150 to 247 (median 190) species. The corresponding figures ranged from 102 to 177 species for tooth biofilm samples and from 78 to 119 species for saliva. Notably, the number of detected species was consistently higher in the tooth biofilm samples than in the saliva samples for both merged and accumulation time-stratified data (Figure 3A). In total, 50 tooth biofilm and 38 saliva species were present in all six participants, and on average, 45% of the detected species were detected in both saliva and the biofilm, but with a significant difference between individuals (from 36 to 52%) (Figure 3B).

Contrasting species abundance in the pooled tooth biofilm data vs. the pooled saliva data in OPLS-DA (*R*^2^ = 0.99, *Q*^2^ = 0.95) reinforced a distinct separation of the tooth biofilm and saliva microbiota communities (Figure 3C). The separation was driven by the enrichment of 11 species in the tooth biofilms: *Actinomyces massiliensis, Actinomyces naeslundii, Campylobacter gracilis, Capnocytophaga granulosa, Corynebacterium durum, Corynebacterium matruchotii, Eikenella corrodens, Fusobacterium nucleatum* subsp. *polymorphum, Leptotrichia goodfellowii, Saccharibacteria* (TM7)[G-1] *bacterium* HMT347, and *S. sanguinis*; and 3 species in saliva: *Catonella morbi, Gemella sanguinis*, and *Peptostreptococcaceae* [XI][G-1] *sulci* (Figure 3D).

### Characteristics of Tooth Region-Specific Microbiota

Microbiota profile diversity was evaluated by comparing the numbers of ASVs, α-diversity determined by the Shannon index, β-diversity (dissimilarity) determined by Bray–Curtis (quantitative), and unweighted (qualitative) UniFrac distances, but reporting is restricted to significant differences.

First, the tooth biofilm diversity of the upper vs. lower jaw and the right vs. left side was compared for each of the 16 tooth sections. No significant differences were found in unpaired or paired testing (all values of *p* > 0.05). Therefore, further characterizations were not done.

**Figure 4:**
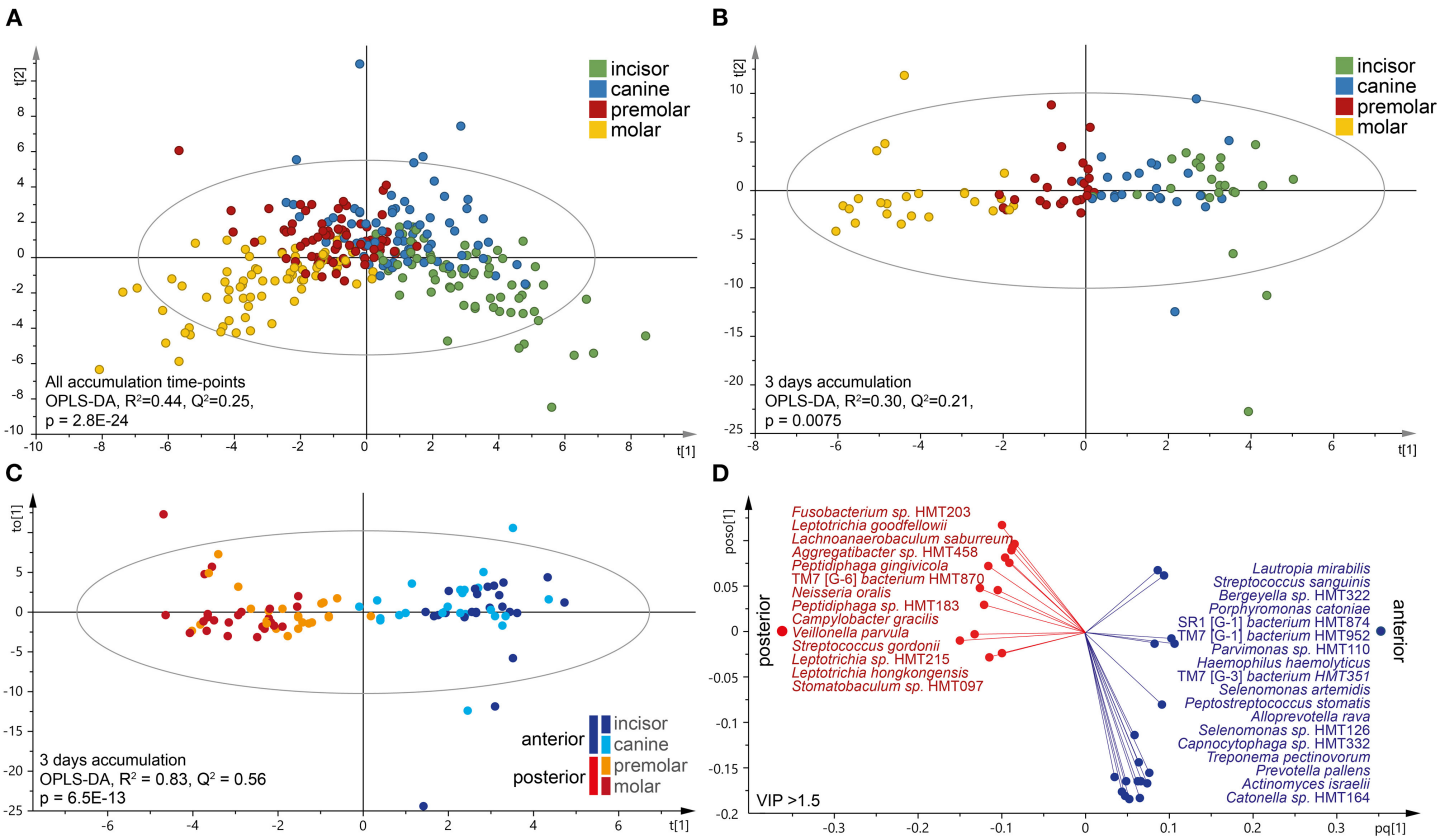
Characteristics of the tooth microbiota in anterior and posterior tooth regions. Multivariate OPLS-DA modeling of **(A)** accumulation-time merged for tooth biofilm microbiota samples or **(B,C)** on day 3 without oral hygiene. In **(B)**, the four regions were modeled as separate entities, and **(C)** is the dichotomous evaluation of anterior vs. posterior regions. OPLS-DA outcomes are presented as score scatter plots by discrimination components *t*[1] and *t*[2]. **(D)** Loading scatter bi-plot includes the most influential (VIP > 1.5) species for separating the anterior (incisor and canine) and posterior (premolar and molar) regions. The horizontal axis displays the *X*-loading (*p*) and *Y*-loading (*q*) of the predictive component, and the vertical axis displays the *X*-loading *p*(*o*) and *Y*-loading *s*(*o*) for the first orthogonal component.

Next, tooth biofilm data from the incisor, canine, premolar, and molar tooth regions, i.e., merged data for the upper and lower and right and left sides for each region, were compared. The OPLS-DA model exhibited a gradual change in the microbiota composition from the most anterior to the most posterior sites (Figure 4A). The separation was emphasized when the analysis was stratified by accumulation day as exemplified for the samples collected after 3 days of accumulation (Figure 4B). The latter scatter plot also showed that the two anterior regions (incisor and canine samples) grouped together, as did the two posterior regions (premolar and molar samples). To increase the power in the analyses, further in-depth comparisons focused on the anterior (incisors and canines) vs. posterior (premolars and molars) groups in accumulation time-stratified analyses.

The linear discriminant analysis effect size analysis (LDA score > 2.0, *p* < 0.05) recognized species belonging to the phylum Proteobacteria and the genera *Bergeyella* and *Neisseria* as being enriched in the posterior regions, whereas the anterior parts were enriched for species in Firmicutes and the genera *Actinomyces, Peptidiphaga, Stomatobactumlum*, and *Veillonella*. Further details, including accumulation time-stratified data, are presented in Supplementary Table S2.

Accumulation time-stratified OPLS-DA models contrasting the anterior and posterior microbiotas yielded similar results (Supplementary Table S3), but with a slightly stronger explanatory (*R*^2^) and predictive (*Q*^2^) power for 3 days without oral hygiene (Figures 4C,D). Thus, the dichotomous model (anterior vs. posterior regions on day 3) confirmed the anterior–posterior separation (*R*^2^ = 0.83, *Q*^2^ = 0.56), but with some regions overlap within the subgroups (Figure 4C). This model identified 32 species as strongly influential for the region separation (VIP > 1.5); *Fusobacterium* sp. HMT203*, L. goodfellowii, Lachnoanaerobaculum saburreum, Aggregatibacter* sp. HMT458, *Peptiphaga gingivicola*, TM7 [G-6] *bacterium* HMT870, *N. oralis, Peptidiphaga* sp. HMT183, *C. gracilis*, and *Veillonella parvula* that were enriched in the posterior parts, whereas the anterior section was enriched with *L. mirabilis, S. sanguinis, Bergeyella* sp. HMT322, *Porphyromonas catoniae, A*. (SR1) [G-1] *bacterium* HMT874*, S*. (TM7) [G-3] *bacterium* HMT952, *Parvimonas* sp. HMT110, *Heamophilus haemolyticus, S*. (TM7) [G-3] *bacterium* HMT351, and *Selenomonas artemidis* (Figure 4D).

### Temporal Trends in Saliva Microbiota Composition

**Figure 5:**
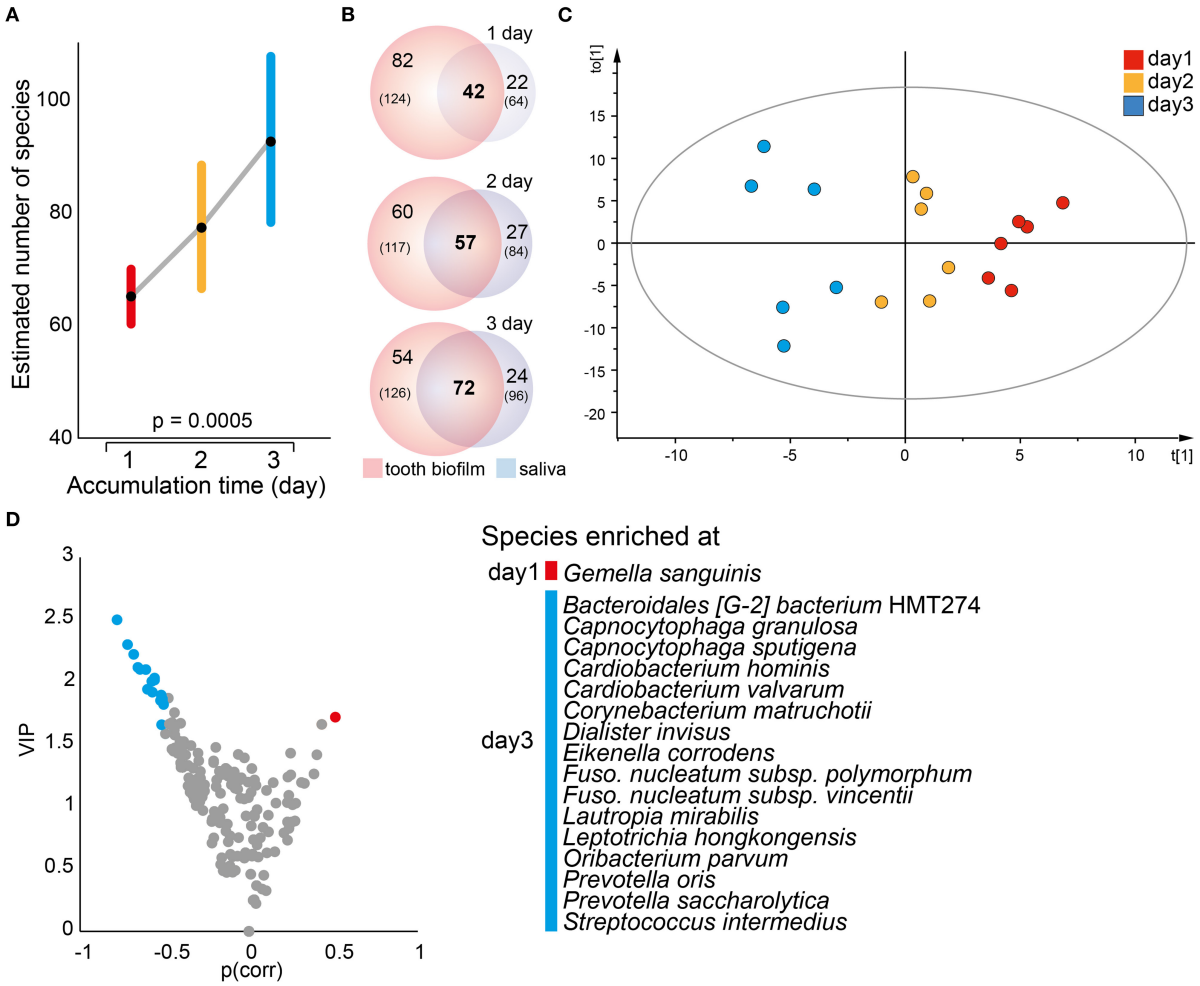
Temporal trends in saliva microbiota composition. **(A)** A mixed linear model of the estimated number of species in saliva samples at each accumulation time. Data are shown as the estimated marginal mean with 95% CIs. **(B)** Venn diagrams illustrating the total number of species detected (*n*) in the pooled tooth biofilm and saliva samples, respectively, whereas numbers in bold represent the species detected in both sample types by increasing accumulation time. **(C)** Multivariate OPLS-DA modeling illustrates a distinct separation of the saliva microbiota composition by discrimination component *t*[1] when increasing the accumulation time from 1 to 2–3 days. **(D)** Volcano plot of *p* {PLS correlation coefficient [*p*(corr) > 0.5] vs. VIP > 1.5} highlights the most influential bacterial species most in separation by accumulation time.

A mixed linear model with 1, 2, or 3 days without oral hygiene as the fixed factor revealed a significant accumulation time-dependent increase in the number of detected species in saliva (*p* = 0.0005, Figure 5A) and the α-diversity (Shannon, *p* = 0.015). In parallel, the number of species, as well as the proportion (% of all detected species), shared between saliva and the pooled tooth biofilm samples increased over time, i.e., the proportions of shared species increased from 29% after 1 accumulation day to 40% after 2 days, and to 48% after 3 days) (Figure 5B). In OPLS-DA, the saliva samples clustered apart for the three accumulation times (*R*^2^ = 0.47, *Q*^2^ = 0.16; Figure 5C), though β-diversity (unweighted Unifrac distance or Bray–Curtis distance) was not significantly different. The model revealed that *G. sanguinis* was enriched on day 1, but *Bacteroidales* [G-2] *bacterium* HMT274, *C. granulosa, Capnocytophaga sputigena, Cardiobacterium hominis, Cardiobacterium valvarum, C. matruchotii, Dialister invisus, E. corrodens, F. nucleatum* subsp. *polymorphum, F. nucleatum* subsp. *vincentii, L. mirabilis, Leptotrichia hongkongensis, Oribacterium parvum, Prevotella oris, Prevotella saccharolytica*, and *Streptococcus intermedius* were enriched on day 3 (Figures 5C,D).

### Temporal Trends in Tooth Biofilm Microbiota Composition

**Figure 6:**
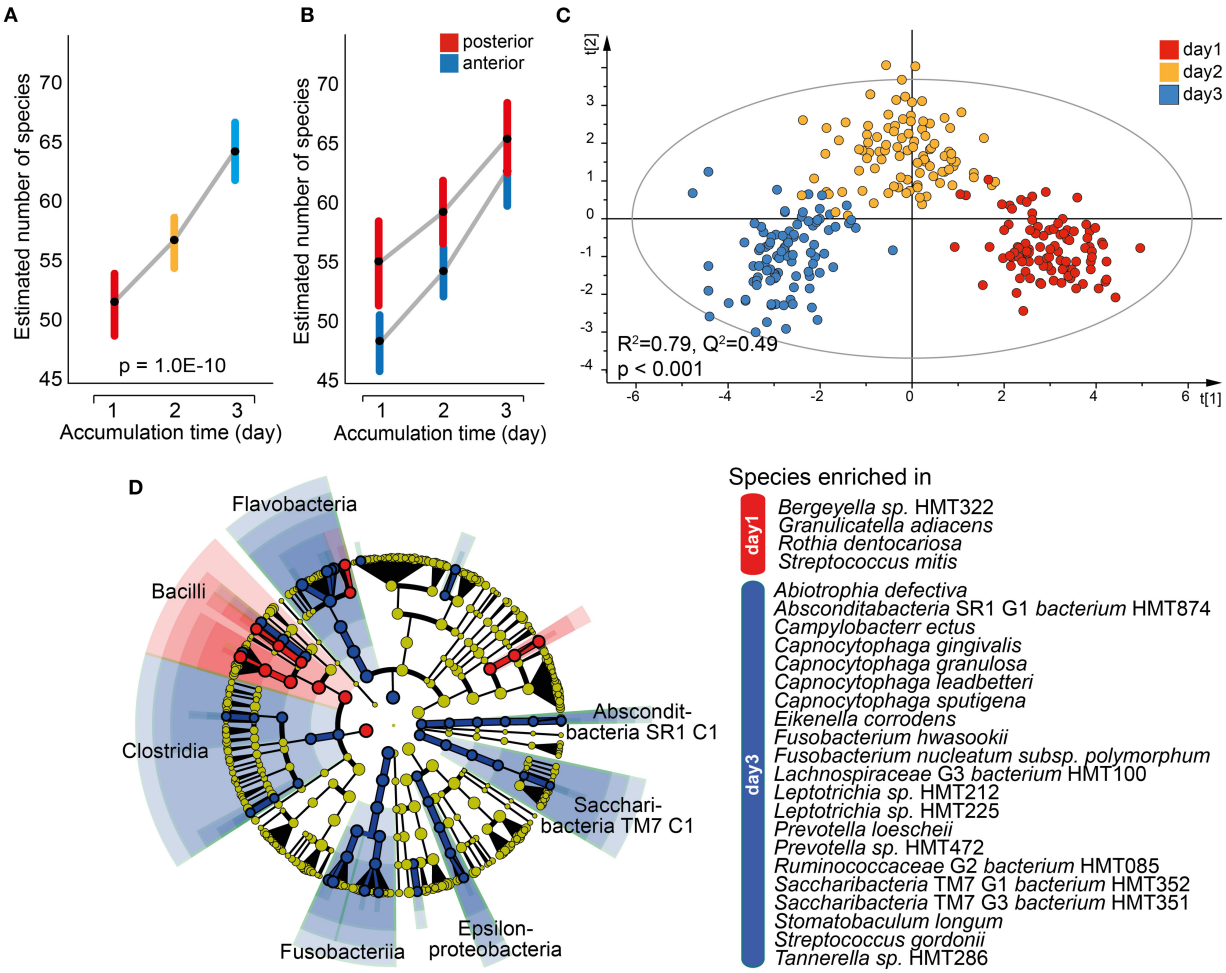
Characterization of the tooth biofilm microbiota by increasing accumulation time. Mixed linear model of individual tooth biofilm samples at each accumulation time for **(A)** all samples or **(B)** anterior (incisor, canine) and posterior (premolar, molar) regions separately. Data are presented as estimated marginal means with 95% CIs. **(C)** Multivariate OPLS-DA modeling of the tooth biofilm microbiota composition collected after 1, 2, or 3 days without oral hygiene, illustrating the separation by accumulation time using discrimination components *t*[1] and *t*[2]. **(D)** Linear discriminant analysis (LDA) effect size analysis (LeFSe) derived phylogenetic differences associated with biofilm maturation and species differing between the three accumulation times (LDA score > 2.0 and *p* < 0.0043, Kruskal–Wallis test, comparing all against all subgroups).

Similar to saliva, a mixed linear model employing data for the 16 individual tooth regions and accumulation time as the fixed effect revealed a significant increase in the mean number of estimated species over time (*p* = 1.0E-10; Figure 6A). The difference was also seen when the anterior and posterior regions were evaluated separately, but with a steeper increase for the anterior region and the means approaching each other on day 3 (Figure 6B). This was not observed in pooled tooth biofilm samples (Figure 3A). The OPLS-DA model with accumulation day as the dependent variable against the taxa swarm of the 16 tooth regions confirmed that the microbiota profile differed by accumulation period (*R*^2^ = 0.79, *Q*^2^ = 0.49; Figure 6C). Here, the α-diversity (Shannon, *p* = 1.1E-19) and β-diversity distance (unweighted Unifrac distance, *q* ≤ 0.0025 and Bray–Curtis distance, *q* ≤ 0.0013) differed significantly between the accumulation time clusters. An LeFSe analysis using the same data set and LDA score > 2.0 (*p* < 0.05) indicated an enrichment of species in the phylum Firmicutes, represented by *Bergeyella* sp. HMT322, *G. adiacens, R. dentocariosa*, and *Streptococcus mitis*, on day 1. On day 3, species in phyla Absconditabacteria SR1, Bacteroidetes, Fusobacteria, and Saccharibacteria TM7, represented by several species in the genera *Capnocytophaga* (*gingivalis, granulosa, leadbetteri*, and *sputigena*), *Fusobacterium* (*hwasookii* and *nucleatum* subspecies *polymorphum*), *Leptotrichia* (*loescheii*, HMT212, HMT225, and HMT472), *S*. (TM7) [G1] *bacterium* (HMT352, HMT351), and *Prevotella* (*loescheii* and HMT472) were enriched (Figure 6D). Further details are given in Supplementary Table S4.

## Discussion

The present study evaluated oral niche-specific microbiota composition (saliva and 16 tooth regions) and the effect of accumulation time (i.e., time without oral hygiene) using V3-V4 amplicons of the 16S rRNA gene. The microbiota profiles in the saliva and tooth biofilm represented the two distinct communities with less than 50% part of a core microbiota. In addition, the participant's microbial profile defined by the phylogenetic profile of the saliva and tooth biofilm communities, respectively, was driven by personal traits rather than accumulation time or sample region. Nevertheless, both accumulation time and sample region were associated with the number of detected species and taxonomic profiles, emphasizing the importance of sample standardization and a conscious decision on sample sources in relation to research question.

Multivariate modeling revealed that each of the participants harbored a distinct profile of sequence variants (ASVs) and taxonomically defined species in the oral cavity, even if saliva from all three accumulation times or tooth biofilm samples from the 16 tooth sites at all three sampling occasions were included in the same models. This supports previous reports on strong individuality, stability, and resilience in the microbiota ecology of the oral cavity [3, 4, 12, 14, 27]. Personal influence has been suggested to be more pronounced for saliva than fecal microbiota based on long-term evaluations of persons in a confined environment [14, 28]. The determinants of individual oral microbiota profiles are suggested to involve both external exposures and host genetics. Thus, studies on twins have found an overall more similar microbiota in monozygotic twins than dizygotic twins, but with a significant bacterial species variation explained by additive genetic effects vs. shared or unshared environmental factors [29]. In contrast, a recent study comparing the transition from mothers to infants and early acquisition of bacteria in the oral cavity in adopted vs. biological children found environmental factors to be exclusive driving forces [30]. This seemingly contradictory information needs to be clarified, including whether the balance between environmental and genetic influences on the oral microbiota differ by age or maturation stage. Nonetheless, the stable community profile may be temporarily upset by extraordinary events, such as antibiotic or irradiation treatments, or permanently modified into a dysbiotic state as reported in frequent or prolonged periods of low pH (i.e., due to sugar intake) [4]. The present study would not capture such a transformation as it evaluated the microbiota within a narrow time window and diet was kept constant over the study period.

A second main finding in this study was the distinct microbiota profiles in tooth biofilm scrapings vs. saliva for both the group and in each of the participants. This difference manifested as a higher number of detected species and diversity in tooth biofilms than saliva regardless of accumulation time, this is in line with some but not with other [20, 27]. Xu et al. [27] showed that dental plaque displayed an increased number of operational taxonomic unit (OTU) compared to saliva samples; however, Simon-Soro [20] displayed a higher number of OTUs in non-stimulated saliva samples, compared to both stimulated and tooth biofilm samples. Potential discrepancies could reflect biofilm collection, a 16S target region, or applied bioinformatic pipelines. The finding of different microbiota profiles in saliva and on teeth is well known from previous studies though the reported species to be a shared or unique variation between the studies [11, 20]. Here, we show that more than 50% of the detected species, on average, were unique to each specimen. Here, the abundance of *S. sanguinis, L. goodfellowii, C. matruchotii, C. durum*, and seven more species distinguished tooth biofilm, and *C. morbi, G. sanguinis*, and *P*. [XI][G-1] *sulci* distinguished saliva. Overall, this finding is in line with previous reports. For example, *S. sanguinis* was reported to be predominantly found in healthy tooth biofilms, *C. matruchotii* to have a nucleating role in tooth biofilm formation, supporting a uniqueness for tooth biofilms [9]. Notably, that a species abundance determining sample separation in multivariate modeling merely reflects its multidimensional variation among the samples and not necessarily that it is among the most abundant species in the sample type. However, an unresolved question is what sample type yields the most valid characterization of the oral microbiota, as well as whether saliva or tooth scrapings should be selected in studies on oral health and if saliva is a valid marker for tooth microbiota. The present study does not allow full elucidation of these questions, but confirms the importance of selecting a sampling site relevant to the research question.

The present study confirms previous characterizations of the oral microbiota with more than 99% of the detected phylotypes being in the phyla Firmicutes, Bacteroidetes, Actinobacteria, Proteobacteria, and Fusobacteria [31, 32]. Overall, the number of phylotypes detected in the oral cavity is reported to be up to 1,000 [2]. From this perspective, a not new [33] but still a notable point is that fewer than 300 species are commonly found in an individual person, which is in line with our finding of 150–247 species in single participants. Notably, there is a significant variation in the number of detected species in the oral cavity, which together with a variation in the number of species detected in both saliva and tooth biofilms emphasizes the individuality in oral microbiota societies.

A commonly targeted research question is what bacterial species are associated with or cause dental caries or periodontal disease [34]. In such studies, the microbiota of resting or chewing stimulated saliva or tooth scrapings from all available tooth surfaces are commonly used as a proxy for one or a few diseased tooth sites, meaning that the profile reflects a majority of healthy sites [35]. Accordingly, the goal should be to collect tooth site-specific biofilm, preferentially before disease development. The first question in a site-specific approach is whether the DNA yield will be sufficient for an analysis. We performed a selection to merge tooth biofilm scrapings from the two adjacent teeth, mainly motivated by the fear that the DNA yield would be insufficient, but also that the number of samples would approach 550 samples for six participants. We found that the average amount of DNA ranges from 0.86 to 11.2 μg/μl for two teeth and 24.1 to 32.9 μg/μl for saliva, and that the amount of extracted DNA was sufficient for sequencing for all individual samples after 24 h of accumulation time. Under the assumption that the total amount of DNA was equal from the two sampled teeth, the estimated yield after 24-h accumulation time would be sufficient for sequencing one tooth in some, though not all participants, as the yield from a single surface is unpredictable. Therefore, studies targeting single tooth sites likely need to rely on longer accumulation times in general.

The number of species increased with increasing accumulation time in saliva and individual tooth site samples, but the latter was not reflected when data for tooth biofilm samples were pooled. It may be hypothesized that the overall species richness in tooth biofilms reaches an upper limit for the individual though species transmission between adjacent sites continue and thereby affect species richness at individual tooth site. Pooled tooth biofilm samples had approximately 40 species more per participant, on average, than saliva samples. Considering that the saliva sampling method has the potential to include more soft tissue-colonizing species than tooth-scraping methods, this is for us unexpected and needs to be evaluated further. It is tempting to speculate that saliva samples does not capture the innermost layers of the tooth biofilm, which may affect its overall richness compared to tooth biofilm samples.

The strengths of the present study include a comparably high number of separate tooth sampling sites, the highly standardized sampling conditions, and the control of potential confounding events during the study period, such as change in dietary habits or health status. The use of PLS multivariate regression may also be a strength as it, in contrast to traditional multiple linear regression, allows covarying *X*-variables in the model; a common feature in bacterial communities. Thereby, the need to run several sequential tests on the same outcome is avoided and the risk for false positive associations is reduced. Applying the OPLS option of PLS provides a stricter evaluation because only predictive and cross-validated values are generated. Thus in summary, OPLS supports a stringent identification of influential variables and the selection of variables for follow-up evaluations with or without variable reduction. The limitations of the study are related to the inclusion of only six participants, which may have reduced the power of species detection, especially for the analyses stratified for single-tooth regions. Consequently, some analyses were based on the merged samples and looked at the anterior vs. anterior sections. Furthermore, the limited number of participants did not allow us to evaluate whether saliva is a valid marker for tooth biofilm, i.e., does the abundance in saliva rank similarly to that of tooth biofilm and, if so, for what species. Still, the number was sufficient for group and trend analyses. In addition, this study has the same limitations as any amplicon-based characterization: a non-optimal resolution at the species level and that species identification is restricted to the quality of the sequence database. Here, we matched our sequences to the eHOMD database, which is a comprehensive curated database for the oral cavity and upper airways with information on 2,074 oral/nasal genomes, representing 529 taxa (http://www.homd.org/).

Though the present study revealed several aspects of the individuality and site-specific differences in the oral microbiota, supporting the existing literature, there are still mechanisms that need to be scrutinized and understood. For example, the relative impact of host genetics vs. environmental factors on the niche-specific oral microbiotas remain controversial, as do the local determinants for tooth region specificity. Tooth structure and morphology and similarities in saliva flush (composition, flow, and muscle activities) are hypothesized to be related to the similarity between the right and left side and upper and lower jaw tooth microbiotas, and also the anterior posterior gradient, but details are limited thus far [36].

## Conclusion

In conclusion, the present study confirms that individuality is the predominant attribute for shaping the oral microbiota communities compared to both the sample type and period of abstaining from oral hygiene. Therefore, a standardized accumulation time may be less important for some research questions aiming at separating individuals. It can also be concluded that the amount of DNA sampled from two teeth is sufficient for microbiota characterization, which allows site-specific characterizations in studies on the development of, for example, caries or periodontitis, instead of pooling all available tooth surfaces, in which the main part of the sample would represent healthy sites.

## Data Availability Statement

The datasets presented in this study can be found in online repositories. The names of the repository/repositories and accession number(s) can be found below: https://www.ncbi.nlm.nih.gov/, PRJNA779698.

## Ethics Statement

The studies involving human participants were reviewed and approved by the study followed the Helsinki Declaration and was approved by the Ethical Committee at Umeå University, Umeå Sweden (Dnr 2017/450-31 with addendum Dnr 2018-199-32). The patients/participants provided their written informed consent to participate in this study.

## Author Contributions

The study was conceptualized by AE, LE, and IJ. LE organized and led the clinical parts of the study. AE performed the sequence analysis, associated bioinformatics, and statistical evaluations. IJ financed the analyses. AE and IJ wrote the draft. All authors contributed and approved the final version.

## Funding

The study was supported by a grant from The Patent Revenue Fund for Research in Preventive Odontology (Grant No. 2018-2021 to IJ).

## Conflict of Interest

The authors declare that the research was conducted in the absence of any commercial or financial relationships that could be construed as a potential conflict of interest.

## Publisher's Note

All claims expressed in this article are solely those of the authors and do not necessarily represent those of their affiliated organizations, or those of the publisher, the editors and the reviewers. Any product that may be evaluated in this article, or claim that may be made by its manufacturer, is not guaranteed or endorsed by the publisher.
